# Optimising a digitally delivered behavioural weight loss programme: study protocol for a factorial cluster randomised controlled trial

**DOI:** 10.1186/s13063-024-08320-5

**Published:** 2024-07-13

**Authors:** Gina M. Wren, Dimitrios A. Koutoukidis, Jadine Scragg, Grace Preston, Marianne Hennessy, Daisy Estephane, Michael Whitman, Susan A. Jebb

**Affiliations:** 1 Nuffield Department of Primary Care Health Sciences, University of Oxford, Oxford, UK; 2 National Institute of Health Research Oxford Biomedical Research Centre, Oxford, UK; 3 Second Nature, London, UK

**Keywords:** Optimisation, Weight loss, Obesity, Overweight, Digital health, Mobile health, mHealth, Multicomponent, MOST framework

## Abstract

**Background:**

Digitally delivered weight loss programmes can provide a convenient, potentially cheaper, and scalable treatment option for people who may need to lose weight. However, outcomes are often inferior to in-person interventions in the long-term. This trial will use principles from the Multiphase Optimisation Strategy (MOST) framework to test whether it can enhance the effectiveness of a commercial digital behavioural weight loss programme. This trial aims to identify an optimised combination of four intervention components to enhance weight loss over a 24-week period. We will also explore which components contribute to improvements in participant retention and engagement with the programme.

**Methods:**

Approximately 1400 adults with a BMI > 21 kg/m^2^ will be enrolled and randomised to one of 16 experimental conditions in a 2^4^ factorial cluster design. The trial will test four intervention components: an introductory video call with the health coach, drop-in webchat sessions with the health coach, goal setting statements, and food diary review and feedback. All participants will receive the core digital behavioural weight loss programme and up to four new intervention components. Participation in the trial will last for 24 weeks. The primary outcome will be weight change at 16 weeks. Other outcomes, measured at 4, 16, and 24 weeks, include programme drop-out and engagement (number of interactions with the three main app functions). Fidelity and acceptability will be assessed using data on component adherence and self-report questionnaires. Decision-making for the enhanced programme will be based on components that contribute to at least a minimal improvement in weight loss, defined as ≥ 0.75kg, alone or in combination with other components.

**Discussion:**

The factorial design is an efficient way to test the efficacy of behavioural components alone, or in combination, to improve the effectiveness of digital weight loss programmes. This trial will test the implementation of the MOST framework in an industry setting, using routinely collected data, which may provide a better way to refine and evaluate these types of interventions in a model of continuous service improvement.

**Trial registration:**

Trial registration: ISRCTN, ISRCTN14407868. Registered 5 January 2024, 10.1186/ISRCTN14407868.

**Supplementary Information:**

The online version contains supplementary material available at 10.1186/s13063-024-08320-5.

## Background

Weight loss, to a body mass index (BMI) within the ‘healthy’ range, has been shown to be beneficial for people living with overweight and obesity but also for people with a BMI below the classification of ‘overweight’ by reducing cardiometabolic risk factors [1]. Population-wide efforts to support people to lose weight are important to reduce the burden of avoidable ill health. The most common intervention for weight loss is dietary and physical activity modification through in-person behavioural support programmes [2]. Although effective, the high costs and resource-intensive nature of such programmes can make them unsustainable for implementation at the population level [3]. New options for effective, accessible, and scalable weight loss interventions are needed.

Digitally delivered programmes provide a convenient, potentially cheaper, and scalable treatment option compared to in-person programmes. A meta-analysis found that compared to in-person interventions, digital interventions led to greater weight loss in the short-term but no difference in the long-term, which could in part be due to poor long-term adherence and retention in digital interventions [4, 5]. Designing and optimising interventions to better retain participants and/or increase weight loss is fundamental to realising the potential of digital interventions.

Mobile health apps are generally an aggregation of intervention components, which are packaged together and offered to participants. Although a standard randomised controlled trial can provide evidence about whether this package works better than a comparator intervention, such designs cannot determine the effectiveness of individual components. Using a randomised controlled trial to evaluate new features individually would require conducting numerous individual studies likely over a long period of time, a process that would lag behind the pace of technological advancement.

The Multiphase Optimisation Strategy (MOST) [6] is an engineering-inspired framework which uses highly efficient randomised experimentation to identify an optimised combination of intervention components before testing this intervention package in a standard randomised controlled trial [6, 7]. Factorial trials offer an efficient design well suited to testing components in a technology-supported obesity intervention [8], as it allows simultaneous testing of the effectiveness of multiple intervention components whilst achieving adequate power using a similar sample size to that needed for a single component [9]. Using the MOST framework, it may be possible to develop more effective behavioural interventions faster, and, when paired with the scalability of digital health interventions, this has the potential to provide considerable public health impact. The present study aims to implement the MOST framework to optimise the effectiveness of a commercial digital behavioural weight loss programme.

### Objectives

The primary objective of this study is to determine if one or more of four new components added to a commercial weight loss programme leads to greater weight loss. The four components to be tested are (1) an introductory video call with the health coach (No vs. Yes), (2) drop-in webchat sessions with the health coach (No vs. Yes), (3) goal setting statements (No vs. Yes), and (4) food diary review and feedback (No vs. Yes). Secondary objectives are to (a) identify which intervention components increase retention and (b) assess whether increased engagement with the programme mediates the association between any observed improvements in weight loss and the four components. The study also aims to explore the fidelity and acceptability of each of the intervention components.

## Methods

**Fig. 1:**
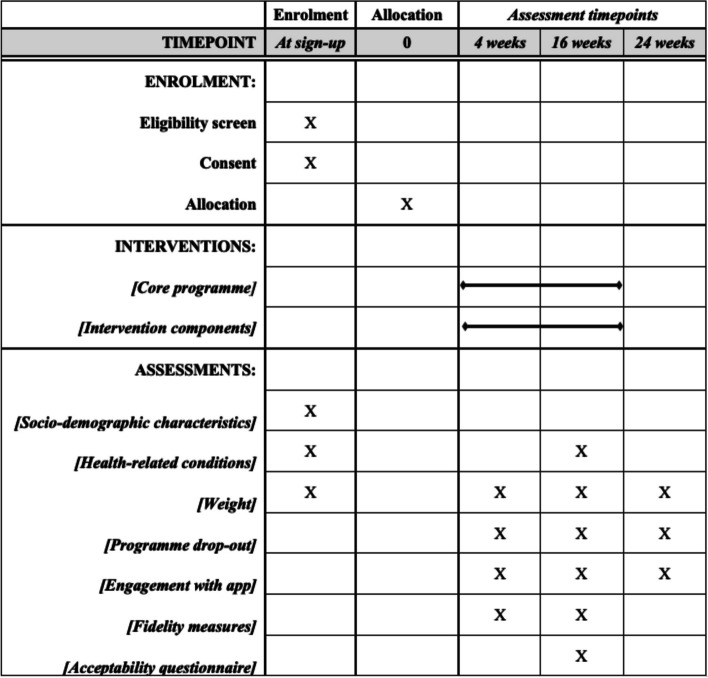
Schedule of enrolment, interventions, and assessments

The Standard Protocol Items: Recommendations for Interventional Trials (SPIRIT) Checklist and figure are provided in Supplementary Material 1 and Fig. 1, respectively

### Intervention component selection

Four intervention components, each with two possible levels, were selected. A list of 93 possible intervention components was generated based on the behaviour change technique taxonomy [10]. This list was condensed to 20 potentially viable components, each supported by empirical literature-based rationale for why it may improve outcomes. This process was informed by expert consultation with academics and healthcare professionals with knowledge of the factors associated with successful outcomes and research into barriers to success as well as consideration of practicality and logistical aspects in collaboration with the commercial partner. The selection of the components was further informed by feedback gained from user interviews with current programme participants to understand key barriers to programme success. Insights from focus groups with the company’s health coaches were used to co-produce the final four intervention components, which helped to translate user feedback into viable components that were practical to introduce in the programme.

**Fig. 2:**
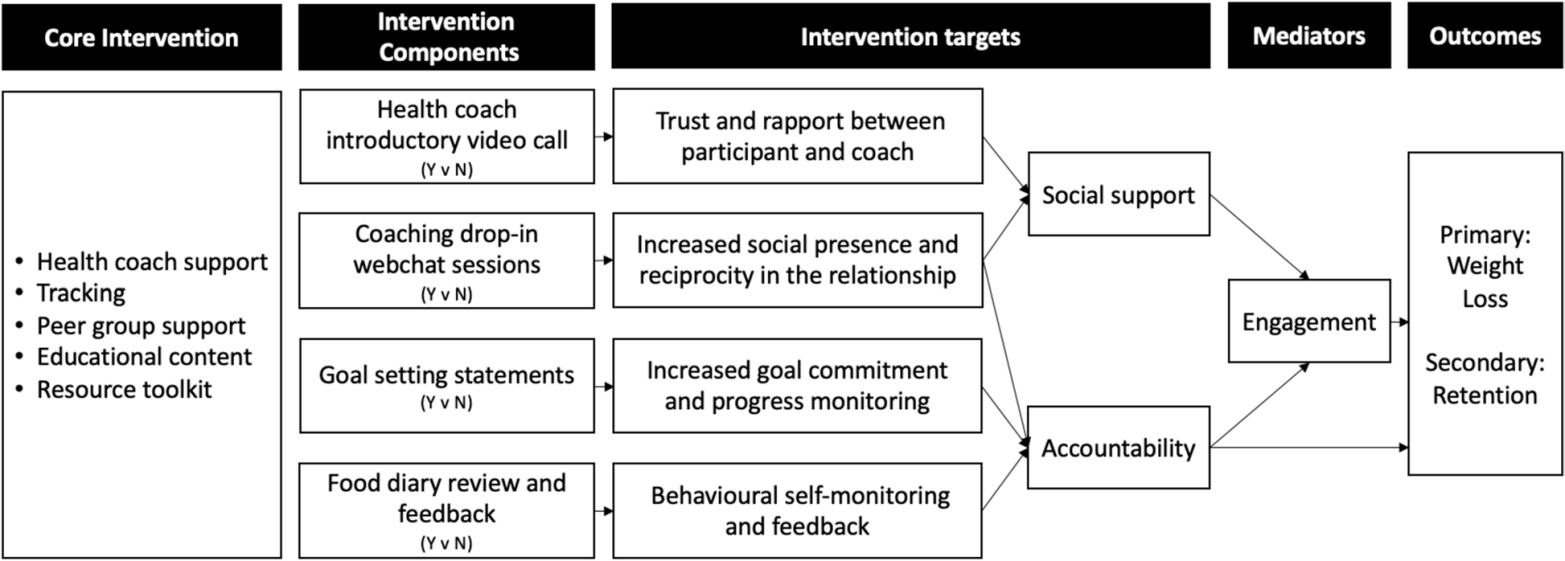
Conceptual model for the optimisation of a digital behavioural weight loss programme, incorporating four new intervention components which target supportive accountability

The four selected components are hypothesised to work together to enhance outcomes and target supportive accountability [11]. They are hypothesised to improve weight loss beyond the core programme, an effect that could, in part, be mediated through enhanced engagement with the programme (Fig. 2). The intervention theory is based on the supportive accountability model whereby individuals are more likely to adhere to an intervention when they feel accountable to a coach that is trustworthy, benevolent, with relevant expertise and whom sets clear process-focussed expectations, establishes goals, and monitors their progress.

### Study design

The study will use a 2^4^ factorial cluster design that tests all possible combinations of four intervention components. The new components will be tested as adjuncts to a core digital behavioural weight loss programme, Second Nature [12]. Second Nature provides a 12-month digitally delivered behavioural intervention designed to support people to have a healthier diet and increase their physical activity eating habits to thus achieve weight loss. The trial began on 8 January 2024 and is expected to run until February 2025.

Participants will be assigned to groups of 28–32 and will undergo the programme within these groups. Approximately 1400 participants, ranging from 1344 to 1536 depending on group size, will participate in the trial. Follow-up in the trial will last for 24 weeks. Each group will be cluster randomised to one of 16 experimental conditions (Table 1).

**Table 1:** Experimental conditions

**Experimental condition**	**Core intervention**	**Health coach introductory video call**	**Coaching drop-in webchat sessions**	**Goal setting statements**	**Food diary review and feedback**
**1**	Y	Y	N	N	N
**2**	Y	Y	N	N	Y
**3**	Y	Y	N	Y	N
**4**	Y	Y	Y	N	N
**5**	Y	Y	N	Y	Y
**6**	Y	Y	Y	N	Y
**7**	Y	Y	Y	Y	N
**8**	Y	Y	Y	Y	Y
**9**	Y	N	N	N	N
**10**	Y	N	N	N	Y
**11**	Y	N	N	Y	N
**12**	Y	N	Y	N	N
**13**	Y	N	N	Y	Y
**14**	Y	N	Y	N	Y
**15**	Y	N	Y	Y	N
**16**	Y	N	Y	Y	Y

### Eligibility

The study aims to enrol adults with a BMI > 21 kg/m^2^, reflecting the usual profile of users of the app. Table 2 presents the key inclusion and exclusion criteria.

**Table 2:** Inclusion and exclusion criteria

**Inclusion criteria**	**Exclusion criteria**
• Adults aged ≥ 18 years • Body mass index (BMI) > 21 kg/m^2^ • Registered to privately access the Second Nature programme during the recruitment window • Able to access the internet with a smartphone or laptop and be willing to instal the Second Nature app on their device	• Past or present diagnosis of an eating disorder • Currently pregnant

### Recruitment

Recruitment is planned to run from January 2024 until August 2024. Participants are recruited to the trial through the routine sign-up process on the Second Nature website (https://www.secondnature.io/). The programme is primarily advertised through various online channels and platforms, such as social media, email marketing, search engine marketing, and affiliate partnerships. All advertisements link directly to the Second Nature website. Participants may also find the website through a personal web search.

### Screening, consent, and baseline assessment

Eligibility criteria will be assessed, and baseline data collected, via Second Nature’s bespoke self-reported health questionnaire which will be completed as part of the sign-up process on the Second Nature website prior to commencement of the programme, with no additional instruction. During the questionnaire, participants will be asked to self-report demographic characteristics (date of birth, and sex), anthropometric measures (body weight and height), health-related conditions (diabetes, physical, and mental health conditions), current pregnancy, and their main priorities and goals for the programme. If the participant is ineligible, a pop-up will explain that they do not meet the eligibility criteria for the Second Nature programme, they will not be able to proceed further, and no identifiable information will be collected.

Participants who meet the eligibility criteria will be able to review and accept the Second Nature Terms & Conditions and Privacy Policy, which involves providing consent to be involved in internal and external research and for their data to be used for academic research purposes (Supplementary Material 2). They will then be able to complete the sign-up process, where they can purchase the programme and input their personal contact details (name, email, and home postcode). After sign-up, participants will receive instructions on how to download the Second Nature app. After downloading the app, participants will also be asked to weigh themselves using their weighing scales and record their baseline weight reading in the app.

Participants have the right to withdraw from the programme, and hence also from the trial, and request that all data that has been collected on them is deleted, with no obligation to give a reason.

### Randomisation

Participants will be allocated to closed groups in which they will undergo the programme. Allocation occurs through an algorithm which assigns participants to groups based on age and sex. Groups consist of male only, female only, and mixed sex groups plus mostly older, mostly younger, and mixed age groups. Only groups that have between 28 and 32 participants will be included in the trial, to ensure a similar number of participants will be included in each experimental condition. Groups in which the number of participants falls outside this range will continue to the standard Second Nature programme. Each eligible group will be cluster randomised to one of 16 experimental conditions. The research team will use computerised randomisation software (MinimPy version 2) to randomly allocate the groups with a minimisation algorithm to balance sex and age across the experimental conditions as much as possible. Randomisation will be performed by a researcher who will not be delivering the intervention and who does not have visibility of the screening process or the allocation of groups.

### Blinding

All participants will be blind to treatment allocation. Due to the nature of the interventions, the health coaches who will be delivering the intervention cannot be blinded, but they will not be involved in data analysis.

### Core intervention

**Fig. 3:**
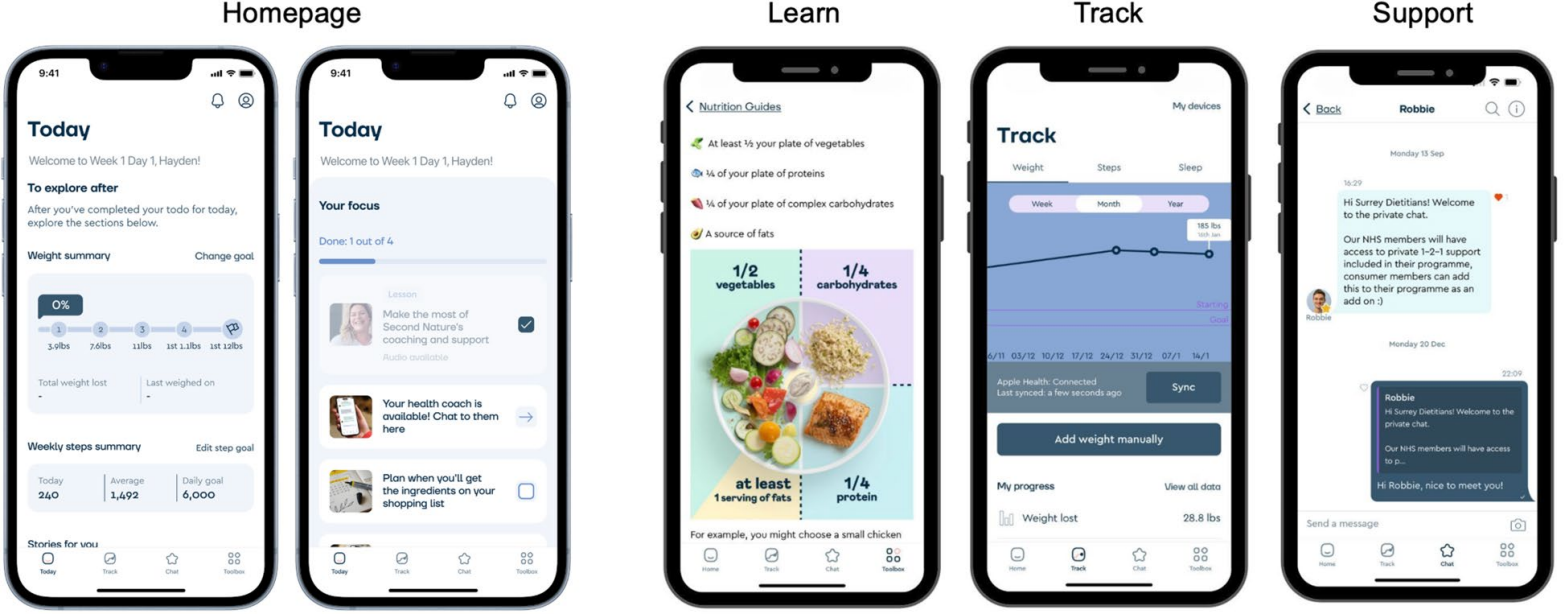
Example Second Nature core programme content

The core intervention, delivered to all participants primarily via a smartphone application, is the ‘core’ phase of the Second Nature programme (Fig. 3). The ‘core’ phase consists of a 17-week behavioural change programme aiming to support people to have a healthier diet and increase their physical activity. Participants will be asked to download the app onto their device, or they can also access the app functions on the Second Nature website and use it throughout the intervention period. Participants will have access to the core Second Nature programme for as long as they continue to pay for it within the 24 weeks of the trial. All content within the app can be translated to other languages as required by the user.

At the start of the programme, each participant receives a hard-copy instructional handbook and recipe book. Second Nature provides a 12-month programme consisting of three 4-month phases: (1) ‘Core’ (weeks 1–17), where participants receive support from their peers and health coach, and acquire knowledge of nutrition and habit formation; (2) ‘Growth’ (weeks 18–34), where participants gain a deeper understanding of their new behaviours and reinforce their new habits; and (3) ‘Maintain’ (weeks 35–52) where participants practice the skills they have learned to maintain their weight loss and continue to lose weight. The ‘core’ 17-week programme consists of mentoring from a health coach, who is either a registered dietitian or nutritionist, who delivers personalised support via a text-based messaging service within the app. The messaging support will be provided both privately and within a group chat of around 30 other participants. The minimum standard frequency for group messages is as follows: weeks 1–4: four messages per week; weeks 5–8: three messages per week; weeks 9–15: two messages per week; weeks 16–17: three messages per week. The group chat is monitored by the health coach, any comments or questions are answered, and further conversation is facilitated where possible. The private chat is only visible to the health coach and the participant and allows participants to ask specific questions on topics such as health-related issues and personalised dietary requirements. All participants within each experimental condition will be in the same group chat and will be assigned to the same health coach. Educational information, delivered by plain text, audio, and videos can be accessed by participants through the app (Table 3). Participants can record and view their weight and step count within the tracking section of the app, which can also be viewed by the participant’s health coach. Participants who have low engagement (defined as < 10 interactions with the app in the previous week) receive automated messages to encourage participation. Whilst weight monitoring is typically self-directed in the standard Second Nature programme, participants in this trial will be prompted by their health coach to weigh themselves at four specific timepoints within the programme (baseline, week 4, week 16, and week 24) to increase follow-up.

**Table 3:** Educational content during each week of the second nature programme

**Week**	**Topic**	**Description**
1	Kickstart week	Introduction to the programme and healthy habits
2	The psychology of eating	The ‘why’ behind certain food choices and overcoming the common obstacles to weight loss
3	Unpacking nutrition	Introduction to nutrition recommendations and common dieting myths
4	The relationship between weight and health	Set point theory, weight loss plateaus, and ways of measuring progress
5	Breaking and making habits	Habit formation, the value of ‘tiny habits’, and stopping unhelpful habits
6	Appetite and cravings	Managing cravings and understanding factors that influence appetite
7	Mindset	Thinking traps, strategies to overcome common thought patterns, and an introduction to body image
8	Stress and emotions	How stress and emotions affect food choices; introduction to mindfulness and emotional eating
9	Exercise	Common barriers to exercise and strategies to incorporate more exercise into daily life
10	Hunger and fullness	The biology and psychology of hunger and fullness and how to be more mindful of fullness cues
11	Nutrition myths	Counting macros, protein intake, alcohol, sweeteners, and reducing risk of heart disease
12	Sleep	The impact of sleep on weight loss and strategies to improve sleep quality
13	Motivation	Types of motivation and how to find and maintain motivation
14	Taking back control	Preparing for challenging situations, social pressure, and impulsive food decisions
15	Reflect and progress	Tools for the next phase of the programme including building self-belief and managing the food environment
16	Accepting thoughts and emotions	Overcoming emotional eating, accepting difficult thoughts, and managing feeling of restriction
17	Moving forwards	Reflecting on the journey so far before moving to the next phase of the programme

During the ‘Growth’ phase, users can still access all features of the app; however, their group will be merged with another group and their health coach may change. The intensity of the programme decreases during ‘Growth’, the message frequency is a minimum of two messages per week, and participation is encouraged less frequently.

The Second Nature programme has been developed based on several behaviour change techniques [10], shown to be effective in diet and physical activity interventions [13], such as self-monitoring, goal setting, feedback, social support, and instruction on how to perform the behaviour. The Second Nature programme has been previously shown to achieve clinically significant weight loss averaging 7.12 kg in those who weighed themselves at 6 months [14, 15] and averaging 6.2 kg after 12 months [16]. Findings also suggest a potential positive association between programme engagement and weight loss [17].

### Intervention components

The intervention components will be delivered as adjuncts to the standard Second Nature programme during the active ‘Core’ phase of the programme only (weeks 1–17).

#### Health coach introductory video call (Y/N)

Telephone coaching has been shown to produce comparable weight loss to in-person coaching [18], and supplementing a web-based programme with coaching calls improved engagement and weight loss outcomes in those that take up the call [19]. Here, participants will be offered either one call from their health coach within the first 2 weeks of the programme or will not be offered an additional call (core programme). Each call will last approximately 20 min. Participants will be sent a link to schedule a timeslot for a call with their health coach. The purpose of the call is for the health coach to introduce themselves, including their qualifications as a registered dietitian or nutritionist, and for the participant to ask any specific questions they may have about the programme. The health coaches will be trained on how to perform the calls and provided with a conversation guide to facilitate discussion.

#### Coaching drop-in webchat sessions (Y/N)

Systematic reviews have shown that frequent contact with a dietitian or therapist is associated with improved weight loss [20, 21]. Participants will either be offered or not offered (core programme) a designated 30-min time slot where their health coach will be ‘live’ in the private chat. This provides a platform for participants to receive timely responses to questions, seek guidance, and receive personalised support as well as providing regular, ongoing accountability. At the end of the first week of the programme, participants will be sent a link to schedule a timeslot to ‘drop-in’ with their health coach for personalised support delivered in the private chat function of the app. Health coaches will be able to schedule up to two participants in each 30-min session. At the scheduled time, the health coach will message the participant in the private chat to begin the session. These drop-in sessions will be offered once a week for the first month and then as required thereafter. At the end of each drop-in session, the health coach will send a booking link where the participant can schedule a suitable time for the next drop-in session. The content of the conversation will be largely led by the participant; however, the health coach will be provided with a conversation guide to prompt conversation if necessary.

#### Goal setting statement (Y/N)

Previous literature has proposed that self-determined motivation, fostered through goal setting, is associated with better weight management [22] and that monitoring goal progress is an effective strategy for promoting goal attainment [23]. Participants will either be asked to complete a goal setting statement alongside regular reflection with their health coach or will not complete a goal setting statement (core programme). Participants will be asked to complete a template in the journal section of the app, where they will be prompted to set an outcome goal (e.g., lose 5 kg by the end of the programme), a process goal (e.g., walk 5000 steps a day), and commit to performing 1–2 actions of their choice (e.g., walk to and from work every day). Participants will then be prompted at the end of each week to answer a series of reflection questions around which actions went well or not so well that week and what they might do differently next week. These reflection questions allow participants to monitor their progress and adjust their strategies accordingly. On a monthly basis, the health coach will prompt the participant to review their goal and ask them whether they wish to adjust the goal. If participants wish to adjust the goal, they will be provided with a new goal setting template to complete in the journal section of the app.

#### Food diary review and feedback (Y/N)

Previous evidence has shown that dietary self-monitoring improves weight loss [24] and that dietary self-monitoring combined with feedback leads to greater weight loss than self-monitoring alone [25]. Participants will be prompted to complete a weekly food diary and offered a review of their food diary entries by their health coach, compared to the core programme in which this occurs on an ad hoc basis. At the beginning of each week, participants will receive a reminder to complete their food diary. At the beginning of weeks 3, 6, and 10, the health coach will message in the group chat offering to review and provide personalised feedback on 1 weeks’ worth of entries for that week. Feedback will be based on Second Nature’s nutritional advice which emphasises the following: (1) Second Nature’s balanced plate model (eat three balanced meals per day, portion size guidelines), (2) base meals on whole foods (limit intake of ultra-processed foods and sugar), (3) meal planning/structure (regular meal timings, healthy snacking), and (4) fluid intake (drink at least 2 litres of water per day, limit alcohol intake, limit caffeine to before midday). Health coaches will review and send feedback to each participant via the private chat function of the app. All food diaries that participants confirm as completed will be reviewed and feedback will be provided within 1 week.

Each of these components are hypothesised to improve weight loss, either directly or in part, through enhanced engagement with the programme, and there might be some synergistic interaction between components. When individuals are provided with components that provide social support (health coach introductory video call or coaching drop-in webchat sessions) as well as components that provide accountability (coaching drop-in webchat sessions, goal setting statements, and food diary review), they will experience adequate supportive accountability, which in turn should increase programme engagement and subsequent weight loss (Fig. 2).

### Public involvement

The intervention components were developed by the researchers, healthcare professionals, dietitians, and the commercial partner. We conducted interviews with current users of the Second Nature programme to understand key barriers to programme success and user preferences, which helped to inform the selection and development of the intervention components. The acceptability and feasibility of the components in terms of perceived helpfulness for weight loss, perceived effort, and whether the components were delivered as planned, will be assessed as part of the process evaluation.

### Outcomes and measures

Outcomes and measures are presented in Table 4.

**Table 4:** Summary of outcomes and measures

**Outcome**	**Outcome measures**
Primary	• Weight change between baseline and 16 weeks
Secondary	• Weight change between baseline and 24 weeks • Drop-out of programme between baseline and 16 weeks • Drop-out of programme between baseline and 24 weeks • Engagement with the programme between baseline and 16 weeks • Engagement with the programme between baseline and 24 weeks
Exploratory	• Weight change between baseline and 4 weeks • Drop-out of programme between baseline and 4 weeks • Engagement with programme between baseline and 4 weeks

#### Weight

Weight data will either be automatically collected using the Bluetooth weighing scales provided at the start of the programme (for those who opt to use them) or can be manually inputted into the app if participants are using their own weighing scales. Data for all participants will be retrieved from Second Nature’s database at baseline and three further time points following the start of the start of the programme: 4, 16, and 24 weeks. Participants will receive a push notification and will be instructed by a message from their health coach in the group chat to self-weigh and log their weight in the app at the end of weeks 4, 16, and 24. Participants who have not submitted a weight reading will be contacted via the private chat 3 days after the follow-up timepoint, via phone call and a further push notification 5 days after the follow-up timepoint. Data for all participants will be retrieved from Second Nature’s database. A single weight reading will be extracted for each time point by searching within a specified time (3–5 weeks for 4 weeks, 14–18 weeks for 16 weeks, and 20–28 weeks for 24 weeks), and the reading closest to the mid-point of each period will be extracted.

#### Programme drop-out

Drop-out will be defined as participants who have chosen to cancel the programme up to and including each follow-up assessment. Drop-out data will be extracted from the app aligning with each follow-up assessment: 0–4 weeks, 0–16 weeks, and 0–24 weeks.

#### Engagement

Engagement data will be collected to assess whether increased engagement with the programme mediates the association between any observed improvements in weight loss and the four components. Following the procedure used previously [17], engagement will be measured as the cumulative total number of interactions with the three main components of the app: ‘Learn’, ‘Track’, and ‘Support’. ‘Learn’ interactions will be defined as the total of number of articles read. ‘Track’ interactions will be defined as the number of times a participant viewed or had a recorded weight or steps reading. ‘Support’ interactions will be defined as the number of messages sent or received in either the private or group chat. Engagement metrics will be extracted from the app at three time periods, aligning with each follow-up assessment: 0–4 weeks, 0–16 weeks, and 0–24 weeks. The exact cut-off time point for each period will be defined based on the date of the extracted single weight reading.

#### Process evaluation measures

##### Acceptability questionnaire

In the penultimate week of the core programme (week-16), participants will be asked to complete a questionnaire, which assesses the acceptability of the overall programme and each component using a 5-point Likert scale. Participants will be asked about the perceived helpfulness of the overall programme to promote weight loss, the perceived helpfulness of each component, and how much effort was associated with each component.

##### Fidelity assessment

We will assess whether the intervention components are delivered as planned by collecting the following measures during the trial period:

- Number of participants attending the health coach introductory call
- Duration of the health coach introductory call for each participant
- Number of participants attending the coaching drop-in sessions each week
- Total number of coaching drop-in session sessions attended per participant, out of a maximum of 17 sessions
- Number of goal setting statements completed per participant, out of a maximum of 16
- Number of reflection statements completed per participant out of a maximum of 16
- Number of food diaries completed per participant out of a maximum of 17
- Number of participant food diaries reviewed by the health coach at each timepoint that the food diary review is offered (weeks 4, 7, and 11)

### Sample size

The available resource allocated to this trial was 48 groups with an average of 30 participants per group. Considering the resource management principle of MOST [7], and given this allocated sample size, we calculated the available power to detect different effect sizes using the R macro %FactorialPowerPlan (Table 5).

**Table 5:** Power calculations

**Cohen’s *d* effect size**	**Difference in weight**	**Power**
0.25	1 kg	92%
0.22	0.88 kg	85%
0.188	0.75 kg	75%

Power for this study is based on weight change from baseline to 16 weeks. Specifically, the study aims to assess which intervention components contribute to a more than minimal improvement in weight loss to identify components for inclusion in the optimised intervention. Our definition of at least a minimal difference in weight loss is ≥ 0.75 kg—alone or in combination with other components—at 16 weeks. We assumed a standard deviation of 4 kg from previous studies [26], which translates to a component effect size of *d* = 0.188. From a decision-priority perspective, this optimisation trial aims to screen components for an optimised intervention that could be tested in a future definitive evaluation [7, 27]. Therefore, the criteria to consider an effect important will be set liberally at *p*< .10, which is not uncommon for this phase of MOST methodology [8].

Based on previous studies, we assumed a 50% retention rate (an average of 15 participants per group) [26] and a cluster size standard deviation of 5 participants, an intraclass correlation of 0.01 and set alpha to 0.1. This sample size provides 75% power to detect a 0.75 kg difference in weight loss and 92% power to detect any differences of 1 kg or larger. Therefore, this study is powered to detect any main effects or interactions of these sizes or larger.

### Statistical Analysis

Descriptive statistics will be used to summarise the demographic characteristics of the sample.

#### Primary objective

Data will be analysed using a linear mixed-effect model with repeated measures, to assess whether each component is associated with weight change across the time points (4, 16, and 24 weeks). Participant ID will be added as a random effect, time and component as fixed effects, and an interaction term of component by time. We will also assess two-way interactions between components by adding appropriate multiplicative interaction terms.

The sensitivity of the analysis to assumptions about missing data will be assessed using appropriate methodology that could include (1) last observation carried forward, (2) baseline observation carried forward, and (3) completer analysis. Planned subgroup analyses will categorise participants into three subgroups based on their baseline BMI: (1) participants with a BMI of 21–25 kg/m^2^, (2) participants with overweight (BMI 25–30 kg/m^2^), and (3) participants with obesity (BMI > 30 kg/m^2^). We will also conduct subgroup analyses by age (based on the median age), sex, tertiles of the index of multiple deprivation (with cut points determined from the study population), and presence of pre-diabetes or type 2 diabetes at baseline.

The decision about which components to select for the optimised intervention (i.e., an intervention that includes only active components) will be based on the main effect and interaction estimates obtained from the primary analysis.

Significance levels will be set at *p* ≤ 0.10. Effect sizes and 90% confidence intervals will be reported for all analyses, given the alpha of 0.10. We will also report the more widely used 95% confidence intervals.

#### Secondary objectives

For the secondary outcome, the proportion of participants that drop-out from baseline to 4, 16, and 24 weeks will be assessed using logistic regression analyses.

We will also explore whether engagement acts as a mediator in the association between components and weight change. Mediation effects will also be tested for significance using the general approach described previously [28]. Engagement will be considered as a mediator if engagement significantly predicts weight and if the group effect is attenuated with adjustment for the engagement variable. The indirect effect and proportion of total effect mediated will also be calculated.

#### Process evaluation

Descriptive statistics will be used to summarise the quantitative intervention component fidelity measures. Descriptive statistics will also be calculated for the individual items on the acceptability questionnaire and overall, for each intervention component.

### Data management

Second Nature will be the data owner and controller. Data collected at assessment timepoints throughout the trial will be stored on their secure servers with trusted 3rd party suppliers, compliant with the United Kingdom General Data Protection Regulation. All identifiable information, which will be provided by the participant to Second Nature, is governed by their privacy policy. Data will be anonymised by Second Nature after study completion and transferred to the researchers at Oxford via a secure University information governance approved method for data transfer. The dataset will be stored on a University secure server with access held by the study team. Within the University server, all data will be stored in password protected folders, accessible only to members of the research team.

### Ethics and dissemination

The investigators will ensure that this study is conducted in accordance with the principles of the Declaration of Helsinki, with relevant institutional regulations, with GCP and General Data Protection Regulations. The study has been reviewed and received ethical approval by the Medical Sciences Interdivisional Research Ethics Committee of the University of Oxford (REF: R89540/RE001). Any substantial changes to the protocol will be submitted as an amendment to the ethics committee and the sponsor. The results of this study will be submitted for publication in peer-reviewed journals, regardless of the outcome. Authorship will be determined in accordance with the International Committee of Medical Journal Editors (ICMJE) guidelines. We will also present our findings at national and international conferences. Findings will be made available to participants and to the wider public using lay summaries on our website (Nuffield Department of Primary Care Health Sciences, University of Oxford), social media, and via the updated trial registry.

### Governance

The Trial Management Group (TMG) will provide oversight of all matters relating to trial management. The TMG will meet monthly or whenever deemed necessary, to review data and the progress of the trial and to troubleshoot and review any issues. The study carries low risk, is of relatively short duration, and has no plausible mechanism to increase the occurrence of any adverse events that would warrant early termination. Therefore, our sponsor (i.e., the University of Oxford) did not require a trial steering committee or a separate data monitoring and ethics committee.

The day-to-day management of the trial will be coordinated by the chief investigator, who will meet weekly with the research assistants who will support the management of the trial. The chief investigator will also meet weekly with the health coaches who will be delivering the intervention and the health coach manager, to discuss progress, review trial conduct, and address any issues surrounding trial delivery. The trial may be subject to audit by the University of Oxford at their discretion, under their remit as sponsor. The process will be independent from investigators.

## Discussion

This trial will conduct the optimisation stage of the MOST framework, to test the effect of four new components on weight loss in a commercial digital behavioural programme. The components are based on a supportive accountability model and were identified using behaviour change theory, combined with insights from participants and the commercial provider.

Application of the MOST framework aligns with the need to produce efficient, effective, and scalable multicomponent interventions, which is particularly relevant in the context of digital weight loss interventions where the effect sizes are relatively small, with significant variability in outcomes and suboptimal engagement rates [29, 30], necessitating a more efficient trial design. The present trial is unique in that it will be implemented within a commercial setting, using routinely collected data. In such contexts, traditional randomised controlled trials that test individual components may fail to address the complexities and keep pace with the rapid changes in the field. The MOST framework is better suited to continuous service improvement, driven by routine data collection and analysis.

Ultimately, this work will contribute to the understanding of which strategies are useful to improve weight loss, engagement, and retention, which is crucial for realising the full potential of digital interventions. If this trial successfully identifies an optimised intervention package, subsequent research stages could involve a definitive randomised controlled trial to evaluate the effectiveness of the optimised intervention.

### Trial status

Protocol version: V1.1, 2 April 2024. Recruitment began on 8 January 2024 and is expected to run until August 2024.

### Supplementary Information

Supplementary Material 1.

## Data Availability

The protocol and statistical code will be available from the authors upon reasonable request. The datasets generated during and/or analysed during the current study are not expected to be made available due to the data being owned by a third party (Second Nature).

## Abbreviations

BMI: Body mass index
MOST: Multiphase Optimisation Strategy
