# Supplementary material for: Optimising a digitally delivered behavioural weight loss programme: study protocol for a factorial cluster randomised controlled trial
Source: Trials. 2024 Jul 13;25:477. doi: 10.1186/s13063-024-08320-5 (PMC11245776; doi:10.1186/s13063-024-08320-5)
Supplement: Supplementary file 1 — Supplementary Material 1. [file 13063_2024_8320_MOESM1_ESM.docx]

## APPENDIX: SECOND NATURE TERMS AND CONDITIONS & PRIVACY POLICY

## Terms and Conditions, including Disclaimers

This document (the "Agreement") is a legally binding agreement between you and Second Nature Healthy Habits Ltd, trading under the name Second Nature, a company registered in England and Wales under number 08511152 ("Second Nature") that governs your use of the online and mobile services associated with Second Nature, including but not limited to, www.secondnature.io and all associated subdomains (the "Website"), and the Second Nature mobile application ("App").

For ease of reference, all the features and functionality of both the App and the Website, together with all its content (whether accessible wirelessly, electronically or downloadable for printing), shall be referred to herein as the "System".

One component of the System is the self-help lifestyle improvement programme with associated expert articles, tools, healthcare tracking technology, and online community (the "Second Nature Service"). All terms governing the System also apply to the Second Nature Service. The System and the Second Nature Service are both owned and managed by Second Nature Healthy Habits Ltd.

Please note that you must read and agree to the terms and conditions of this Agreement before you use the System and/or the Second Nature Service. If you do not agree to the terms and conditions of the Agreement, you may NOT use the System nor may you use the Second Nature Service.

The terms "we", "us", "our" and "ours" when used in these terms mean Second Nature, which includes any parent company, subsidiaries, branches or affiliates under common ownership or control of Second Nature. The terms "you", "your" and "yours" when used in these terms mean any user of the System.

**1. Subscriptions & Terms**

Subscription and auto-renewal. Your subscription to the Second Nature service includes enrollment into an ongoing/recurring payment plan. Your subscription will automatically renew at the end of the disclosed billing period or 14-day trial; this will be monthly or annually according to your chosen plan, unless cancelled in accordance with the instructions for cancellation below. Payment will be charged to your chosen payment method at confirmation of purchase and at the start of every new billing period, unless cancelled.

We reserve the right to change your pricing and, in the event of a price change, we will notify you thirty (30) days in advance of the change by sending an email to the email address you have registered for your account. If you do not wish to accept a price change, you may cancel your subscription in accordance with the instructions included in that email and below. If you do not cancel your subscription after the price change takes effect and prior to the start of your new subscription period, your subscription will be renewed at the price in effect at the time of the renewal, without any additional action by you, and you authorise us to charge your payment method for these amounts.

Cancellation and Refund Policy. You can cancel your subscription at any time before the end of your current billing period or 14-day trial. Cancellation will take effect at the end of your current billing period or 14-day trial. To cancel your Second Nature subscription follow these [steps](https://help.secondnature.io/en/articles/5184089-cancellation-policy). Cancellations will only take effect at the end of your current billing period (or end of your 14-day trial), and you will still be able to access Second Nature until then. We do not refund or credit for partially used billing periods. Your 14-day trial officially starts on day 1 of your selected start date.

Payment details. We will keep your detailed payment information, such as credit card number and expiry date, on file. You are responsible for keeping your payment details up-to-date by contacting customer support. You authorise us to continue to charge your card using the updated information. If a payment is not successfully authorised due to expiration, insufficient funds, or otherwise, we may suspend or terminate your subscription by giving you notice. You also agree that we may charge your payment method on file if you decide to restart your Second Nature subscription, unless you specify an alternative payment method when you restart your subscription.

Tech Package. If you have selected the ‘tech package’ add-on and wish to return it, you have 14 days from the day you receive your goods to inform customer support. You then have a further 14 days from the date you notify customer Support to ensure the goods are returned to receive a full refund. You will be given a free returns label to send back the healthcare technology that you have been provided with. Please do not return the Tech package to our office address. Your free return label will include the correct address. Upon receipt of the returned hardware you will be offered a full refund if the weighing scales are not materially damaged (i.e. they can be reused for another customer). If the weighing scales are materially damaged then you may be liable for the full cost paid.

**2. Contract Information**

By using the System you confirm that you are 18 years of age or more, that any registration information that you submit to Second Nature is true, accurate and complete, that you will update such information in order to keep it current, that you have read and agree to the terms and conditions contained in this Agreement, our Privacy Policy and our Posting Policy (both of which are hereby incorporated into this Agreement) and that you shall be legally bound by such terms subject to all applicable laws and regulations.

**3. Understanding Second Nature**

You acknowledge and agree that the Second Nature Service is a personalised self-help system designed to help you improve your own wellbeing and that if you choose to access the Second Nature Service you are solely responsible for deciding which of the suggested techniques you put into practice and how to apply those techniques.

You also acknowledge and agree that Second Nature is not a medical organisation and that the Second Nature Service is not intended to diagnose, treat or otherwise address any medical problem. The material on the System, whether posted by Second Nature employees, or other users, is provided for your information and not as medical advice and should not be seen as a replacement for consultation with a doctor or other qualified care professional. You are urged to seek the advice of a doctor before beginning any weight loss programme. If you receive advice from a doctor or other qualified medical professional which conflicts with anything contained in the System, then the former should take precedence. The Second Nature Service, the App and this Website are not intended for use by minors, or pregnant women. Individuals with any type of health condition are specifically warned to seek professional medical advice prior to initiating any form of weight loss programme.

**4. Grant of right of use**

The System is only available to either individuals who enter their card details, PayPal details, or who have had the Service commissioned for them by a third party partner (e.g. NHS England, a Clinical Commissioning Group, or their employer) ("Paid for Services"). Unless otherwise specified, we grant you a limited, non-exclusive, non-transferable, revocable right to make personal non-commercial use only of that content and those features of the System and Second Nature Service in respect of which you or the third party have paid all applicable rental fees and charges, provided that you comply fully with the provisions of this Agreement.

**5. Changes to the Agreement**

Second Nature reserves the right to vary this Agreement from time to time by amending this page. Registered user's will be notified of changes by the email address we have for you on file. Any material amendments intending to bind an existing user of the System shall become effective 7 days after the email.

**6. Your health and Second Nature**

You are urged and advised to seek the advice of a doctor before beginning any lifestyle improvement program. If you have any other concerns or questions about your health or medicines, you should always consult an appropriate healthcare professional. Be cautious about embarking on vigorous exercise if you have any serious medical condition, including (but not limited to) back pain, high blood pressure or heart disease, or if you are pregnant. Always seek professional medical advice when contemplating any changes in your prescribed medicines.

This programme is intended as a lifestyle change guide and is therefore not classified as a medical device, meaning the programme should not replace any existing recommendations that have been given by a health care professional. The content is not intended to be a substitute for professional medical advice, support, diagnosis, or treatment.

Those with suspected/diagnosed eating disorders may be deemed ineligible to partake in the programme. Current best practice to diagnose and treat eating disorders supports the use of in-person therapy, which goes beyond our scope of practice.

Second Nature is not suitable for women who are pregnant due to the different nutritional requirements to support growth and development during pregnancy. Women who are breastfeeding are eligible to partake in the programme. However, as this remains an important time for the growth and development of a child, it is essential that all nutritional requirements are met. Therefore, if you are breastfeeding, you will need to inform your health coach to ensure they are able to support you in the best way possible.

A team of nutritionists have provided advice for dietary information provided on the System and contained within the Second Nature Service. Care has been taken to confirm that the information presented by authors is accurate and describes generally accepted practices. If we find, or are alerted to, a mistake, we will correct it as quickly as possible. However, to the fullest extent permitted by applicable laws, the authors, editors and publishers are not responsible for errors or omissions, or for any consequences arising from application of the information on the System, and make no warranty, express or implied, with respect to the contents, completeness or accuracy of the material published.

**7. Privacy**

We process information about you in accordance with our Privacy Policy. By using the System, you consent to such processing and you warrant that all data provided by you is accurate.

To access the Second Nature Service, you may be required to register personal information and establish unique usernames and passwords. You are responsible for maintaining the confidentiality of any passwords, and are fully responsible and or liable for all activities that occur under your password and username, whether by you or by anyone else using your identity. Should your password be compromised by breaches of security such as, but not limited to, loss, theft and unauthorized disclosure, it is your responsibility to immediately notify us of the need to change or deactivate the password. Until we are so notified you will remain liable for any unauthorized use of your account.

The System contains functionality that allows you to upload content in a public area (including, but not limited to, your online group). By submitting such content, you agree that such submission is non-confidential for all purposes. Additionally you automatically grant us, or warrant that the owner of such content or intellectual property has expressly granted us, a royalty free, perpetual, irrevocable, world-wide nonexclusive license to use, reproduce, create derivative works from, modify, publish, edit, translate, distribute and display the content in any media or medium, in any form, format or forum now known or hereafter developed. If you wish to keep any such information private, do not submit them to a public area or email us as soon as practicable. You are solely responsible for any content you submit to a public area, the consequences of posting such content, and your reliance on any other content found in your online group.

**8. Intellectual Property Rights**

The layout, design, content and graphics on the System and the Second Nature Service are protected by UK and other international copyright laws. Unless expressly permitted in writing and other than printing a small proportion of content or displaying this on your screen or both, (strictly for your personal non-commercial use), no part of the System may be reproduced or stored in any medium, including but not limited to a retrieval system, or transmitted, in any form or by any means (electronic, mechanical, photocopying, recording, broadcasting), nor shown in public. You may not create any derivative work or make any other adaptation, without our prior written consent. You must not modify the copies you have displayed or printed in any way and you may not use any illustrations, photographs, videos or audio sequences or any graphics separately from any accompanying text.

If you print off or download any material from the System in breach of these terms of use, your right to use the System and the Second Nature Service will cease immediately and you must at our option return or destroy any copies of the materials you have made. All rights not expressly granted in these terms or any express written license, are reserved. For all other uses of our content or images you must contact us and obtain our prior written permission.

**9. Prohibited uses**

You agree that you will not use the System to:

- — Upload, post, email or otherwise transmit any content or materials that are unlawful, harmful, threatening, abusive, harassing, tortious, defamatory, vulgar, obscene, libelous, invasive of another's privacy, hateful, or racially, ethnically or otherwise objectionable, at our sole and absolute discretion;
- — Harm minors in any way, or solicit or otherwise attempt to gain any information from a minor;
- — Impersonate any person or entity, including, but not limited to, any user of this System, a director, officer, employee, shareholder, agent or representative of Second Nature or our affiliates, or any other person or entity, or falsely state or otherwise misrepresent your affiliation with Second Nature, our affiliates or any other person or entity
- — Forge headers or otherwise manipulate identifiers in order to disguise the origin of any Postings or other materials transmitted to or through the System
- — Upload, post, email or otherwise transmit any materials that are not your own, or that you do not have a right to upload, post, email or otherwise transmit under any law or under contractual or fiduciary relationships (such as insider information, proprietary and confidential information learned or disclosed as part of employment relationships or under nondisclosure agreements)
- — Upload, post, email or otherwise transmit any content or other materials that infringe upon any patent, trademark, trade secret, copyright, right of privacy or publicity or other proprietary rights of any party
- — Upload, post, email or otherwise transmit any unsolicited or unauthorized advertising, promotional materials, "junk mail," "spam," "chain letters," "pyramid schemes," or any other form of commercial solicitation except in the areas, if any that are specifically designated for such purpose
- — Upload, post, email or otherwise transmit any content or other materials that contain software viruses or any other computer code, files or programs designed to interrupt, destroy or limit the functionality of any computer software or hardware or telecommunications equipment
- — Disrupt the normal flow of dialog, cause a screen to "scroll" faster than other users of the System are able to type, or otherwise act in a manner that negatively affects or otherwise diminishes the quality of another user's experience of this System
- — Interfere with or disrupt the System or servers or networks connected to the System, or disobey any requirements, procedures, policies or regulations of networks connected to the System
- — Intentionally or unintentionally violate any applicable laws and/or regulations
- — "Stalk" or otherwise harass another user of the System and/or any employee of Second Nature
- — Solicit, collect or post personal data or attempt to solicit, collect or post personal data about other users of the Website from the Website (including user names or passwords) or about any other third party, and/or:
  1. — Access or attempt to access another user's account without his or her consent.
  2. — Knowingly transmit any data, send or upload any material that contains viruses, Trojan horses, worms, time-bombs, keystroke loggers, spyware, adware or any other harmful programs or similar computer code designed to adversely affect the operation of any computer software or hardware.

You also agree not to:

- — Reproduce, duplicate, copy or re-sell any part of the System in contravention of the provisions of this Agreement.
- — Resell access to the System.
- — Frame the System or any part of it.
- — Copy any material obtained from the System to peer to peer networks or other web sites or web services.
- — Access without authority, interfere with, damage or disrupt any part of the System; any equipment or network on which the System is stored; any software used in the provision of the System; or any equipment or network or software owned or used by any third party.

**10. Breach of the Agreement**

We will determine, in our absolute discretion, whether there has been a breach of this Agreement through your use of the System. When a breach of this Agreement has occurred, we may take such action as we deem appropriate, which may include all or any of the following actions:

- — Immediate, temporary or permanent withdrawal of your right to use the System and/or the Second Nature Service.
- — Immediate, temporary or permanent removal of any posting or material uploaded by you to the System and/or the Second Nature Service.
- — Issue a warning notice to you to immediately cease and/or remedy such breach and following receipt such action should be taken immediately.
- — Legal proceedings against you for reimbursement of all costs on an indemnity basis (including, but not limited to, reasonable administrative and legal costs) resulting from the breach.
- — Any further legal action against you.
- — Disclosure of such information to law enforcement authorities as we reasonably feel is necessary.

We exclude liability for actions taken in response to breaches of this Agreement. The possible actions we may take on breach of this Agreement are not limited to those described here, and we may take any other action we reasonably deem appropriate.

**11. No warranty**

The use of the System and Second Nature Service (including but not limited to their content and features) is at your own risk. The System is provided on an "as is" and "as available" basis. To the extent permitted by applicable law, Second Nature gives no warranty, express or implied, as to the quality, content and availability or fitness for a specific purpose of the System or the Second Nature Service or to the accuracy of the information contained in any of the materials on the System or the Second Nature Service. Second Nature shall not be liable to any person for any loss or damage of any kind, which may arise, directly or indirectly from the use of or inability to use any of the information contained in any of the materials on the System. There is no guarantee of availability of information on the System at any time, nor that it is up to date or error-free. As with any purchase of a product or service through any medium or in any environment, you should use your judgment and exercise caution where appropriate. No advice or information whether oral or in writing obtained by you from Second Nature shall create any warranty on behalf of Second Nature in this regard. Certain jurisdictions do not allow the exclusion or disclaimer of certain warranties. Accordingly, some of the above disclaimers may not apply to you. Your statutory rights as a consumer, if any, are not affected hereby.

**12. Limitation of liability**

To the fullest extent applicable permitted by applicable laws, Second Nature, its affiliates, officers, directors, employees, licensors or any third parties exclude liability for any direct, indirect, incidental, special or consequential damages (including but not limited to any loss of data, service interruption, computer failure or pecuniary loss, loss of income or revenue, loss of business, loss of profits or contracts, loss of anticipated savings, loss of data, loss of goodwill, wasted management or office time and for any other loss or damage of any kind, however arising and whether caused by tort (including negligence), breach of contract or otherwise, even if foreseeable) arising out of the use of or inability to use the System or Second Nature Service, even if you have advised Second Nature about the possibility of such loss, and including any damages resulting therefrom. Commentary and other materials posted on the System by other users are not intended to amount to advice on which reliance should be placed. We therefore, to the fullest extent permitted by applicable laws, disclaim all liability and responsibility arising from any reliance placed on such materials by any visitor to the System, or by anyone who may be informed of any of its contents. If any part of this limitation of liability is found to be invalid or unenforceable for any reason, then the aggregate liability of Second Nature (including any of its affiliates, service providers and licensors) under such circumstance for liabilities that otherwise would have been limited, shall not exceed the maximum single purchase price of the Second Nature Service. Any claims arising out of or in connection with your use of the System must be brought within one year of the date of the event giving rise to such action occurred.

Nothing in this provision affects our or our contractors' liability for death or personal injury arising from our (or their) negligence nor our (or their) liability for fraudulent misrepresentation or misrepresentation as to a fundamental matter nor any other liability which cannot be excluded or limited under an applicable law.

**13. Technology limitations and modifications**

Second Nature will make reasonable efforts to keep the System operational. However, certain technical difficulties, maintenance, or any other cause beyond our reasonable control (including (but not limited to) failure of the internet, natural disaster, labor shortage or dispute, or governmental act) may, from time to time, result in interruptions. Second Nature reserves the right at any time and from time to time to modify or discontinue, temporarily or permanently, functions and features of the System with or without notice. You agree not to hold us liable for any such failure or delay in performance and we agree to make all reasonable efforts to restore the System (or any part thereof) as soon as practicable. Following any disruption within Second Nature's reasonable control we shall extend your access to the Paid for Services for at least the duration for which those Paid for Services were inaccessible on written request to the below address.

**14. Third parties**

Certain hypertext links in this site may lead to other third party websites, which are not under the control of Second Nature. When you activate any of these you will leave the System and Second Nature has no control over, and will accept no responsibility or liability, for the material on any website which is not under the control of Second Nature. Second Nature does not make any representations or give any guarantee or warranties of any kind, expressed, implied or otherwise about the content of on any external website link.

**15. Indemnity**

You agree to indemnify and hold Second Nature and its officers, directors, employees and licensors harmless from any claim or demand (including but not limited to reasonable legal fees) made by a third party due to or arising out of or related to your violation of the terms and conditions of this Agreement or your violation of the System (and all related materials) or any applicable laws, regulations or third party rights.

**16. Assignment by Second Nature**

Second Nature may assign (or otherwise transfer) this Agreement or any part of it without restrictions. You may not assign (or otherwise transfer) this Agreement or any part of it to any third party.

**17. Entire agreement**

This Agreement (incorporating these terms and the Privacy Policy and the Posting Policy as may be updated from time to time) constitutes all the terms and conditions agreed upon between you and Second Nature and supersede any prior agreements in relation to the subject matter of this Agreement, whether written or oral. Any additional or different terms or conditions in relation to the subject matter of this Agreement in any written or oral communication from you to Second Nature are void. You agree and accept that you have not accepted the terms and conditions of this Agreement in reliance of or to any oral or written representations made by Second Nature not contained in this Agreement.

**18. Severability and waiver**

If any provision of the Agreement is held by a court of competent jurisdiction to be unlawful, void or for any reason unenforceable, such provision shall be changed and interpreted to best accomplish the objectives of the original provision fully permitted by law, and the remaining provisions of the Agreement shall remain in full force and effect.

If we fail to insist that you perform any of your obligations under these terms, or if we do not enforce our rights against you, or if we delay in doing so, that will not mean that we have waived our rights against you and will not mean that you do not have to comply with those obligations. If we do waive a default by you, we will only do so in writing, and that will not mean that we will automatically waive any later default by you.

**19. Term and termination**

This Agreement is between you and us only and will become effective when you start using the System and will remain effective until terminated by you or Second Nature. Second Nature reserves the right to terminate this Agreement or suspend your Second Nature account at any time in case of unauthorized, or suspected unauthorised use of the System whether in contravention of this Agreement or otherwise. If Second Nature terminates this Agreement, or suspends your Second Nature account, for any of the reasons set out in this section, Second Nature shall have no liability or responsibility to you whatsoever.

**20. Governing law and disputes**

The System is controlled by Second Nature from its offices in the United Kingdom. Access to, or use of, the System, including the Second Nature Service and any related information and materials, may be prohibited by law in certain jurisdictions. You are responsible for compliance with all applicable laws of the jurisdiction from which you are accessing the System. We make no representation that the information contained herein is appropriate or available for use in other locations.

This Agreement and any contract between us, whether for use of the System or other purpose, and any non-contractual obligations (if any) arising out of or about these terms and conditions or any such contract will be governed by English law. The parties agree that the courts of England have exclusive jurisdiction to settle any dispute arising out of or about this Agreement (including a dispute regarding the existence, validity or termination of this Agreement or any non-contractual obligation arising out of or about this Agreement). Notwithstanding the foregoing, we reserve the right to bring legal proceedings in any jurisdiction where we believe a breach of this Agreement has originated.

**21. Survivorship**

The following provisions shall survive termination of this Agreement: Clause 8 (Intellectual Property), Clause 12 (Limitation), Clause 15 (Indemnity), Clause 17 (Entire Agreement), Clause 18 (Severability and waiver) and Clause 20 (Governing Law and Disputes).

**22. Peer referrals**

With the application of your Second Nature peer referral code, you (the ‘referring user)’ may be entitled to credit towards your Second Nature account when the person joining through your Second Nature referral code (the ‘referred user’), successfully completes their first two weeks with the programme and does not terminate their subscription prior to. At this point, and if applicable, the credit will be added to your Second Nature balance and will be visible in the mobile app.

**23. Contact**

You can contact us via the following details:

- — Email: hello@secondnature.io
- — Post: Second Nature, Scale Space 58 Wood Ln, London W12 7RZ

Please note that we are unable to answer any enquiries requesting medical advice. Such enquiries should be addressed to an appropriate, qualified health practitioner.

Effective Date: This document is effective as of April 13th 2022.

## Appendix D: Privacy Policy

Second Nature Healthy Habits Ltd, trading under the name Second Nature, ("we") are committed to protecting and respecting your privacy. We are registered with the UK Information Commissioner's Office as a Data Controller (Reg No. ZA148098), and have in place a comprehensive Company data protection policy and code of practice.

This Privacy Policy ("Policy") (together with our Terms, which can be accessed at https://www.secondnature.io/terms, and any other documents referred to on it) sets out the basis on which any personal information we collect from you, or that you provide to us, will be processed by us and how you can get access to this information. If you are located in the European Economic Area (“EEA”) or the United Kingdom (UK), “personal information” means any information relating to an identified or identifiable individual. Please review it carefully.

1. **Purpose of this Policy**

Second Nature provides you (the "User") with access to the online and mobile services including but not limited to, secondnature.io and all associated subdomains (the "Website"), the Second Nature mobile application (the "App"), and any provided healthcare tracking technology, collectively the "System". Our privacy policy is designed in accordance with numerous national and international regulation frameworks, including (but not limited to) the General Data Protection Regulation (“GDPR”), the UK Data Protection Act 2018 and the UK General Data Protection Regulation (“UK GDPR”).

1. **What personal information do we hold and how we get it**

We may collect and process personal information provided by filling in forms on the Website or App, including personal information provided during completion of surveys and other online tools, posting of comments in the Community or requesting further services, and when you report a problem with our System. If you contact us, we may also keep a record of that correspondence. Second Nature also collects and processes personal information with the health tracking technology provided as part of the System, such as wireless weighing scales (which track your weight) and activity trackers (which track your steps and sleep).

Throughout your use of the System we may collect personal information such as: personal demographics information (including but not limited to first and last names, date of birth or age, address, email, phone number); lifestyle or health data (referred as “special category data” and includes height, weight, body mass index, ethnicity, smoking status); other personal health profile information and details of your visits to the System and the resources that you access (including, but not limited to, traffic data, location data, weblogs, other communication data, and the resources that you access).

Your personal information (steps per day and weight) may also be collected via Apple HealthKit or Google Fit upon installing our iOS and Android apps. This consent will be explained and obtained from you within the app and you may revoke this access at any point within your phone's operating system settings.

Your personal information, including your health data (referred as “special category data” and includes height, weight, HBA1C level, BP level, cholesterol level, PAM score) may also be provided to us by an electronic patient record, referral or through a secure online platform in order to refer you to the System, e.g. provided through your GP, local NHS service or our partner REED. If this is the case, the relevant party will ask for your explicit consent .

We collect as well technical information and analytics from you concerning your use of the System, including but not limited to pages visited, links clicked, non-sensitive text entered, mouse movements, system and operating system type and version, browser or app version, time zone setting and usage of our iPhone and Android apps.

1. **IP addresses and cookies**

We may collect personal information about your device, including where available your IP address, operating system, browser type and screen size for use in system administration, to tailor your experience of the System, provide you with customer support and to report aggregate information internally.

For the same reason, we may obtain personal information about your usage of the System by using a cookie file which is stored on the hard drive of your device. Cookies help us to give you a smooth user experience, improve the System and deliver a better and more personalized service. They enable us to: recognize you when you return to our site; maintain personal information you have entered (e.g. during completion of a survey); speed up your searches; estimate our audience size and usage pattern; store information about your preferences; and allow us to customize our site according to your individual interests.

Both Second Nature and our third-party vendors, including Google, may use first-party cookies (such as the Google Analytics cookie) to inform, optimize, and serve ads based on your past visits to the Website on sites across the Internet (also known as 'remarketing'). If you would like to opt out of this you can do so via your Google Ads Preferences Manager.

Below is an overview of the types of cookies we and third parties may use to collect information.

- — Strictly necessary cookies. Some cookies are strictly necessary to make the services available to you. We cannot provide you with the services without this type of cookies.
- — Functional cookies. These are used to recognize you when you return to the services. This enables us to adapt our content for you, and remember your preferences (for example, your choice of language or region).
- — Analytical or Performance cookies. We also use cookies for website and app analytics purposes in order to operate, maintain and improve our services. We may use our own analytics cookies or use third party analytics providers such as Facebook, FOSPHA, HotJar, Metabase, Mixpanel, Twitter, Taboola and VWO to collect and process certain analytics data on our behalf. These providers may also collect information about your use of other websites, apps, and online resources. You can opt out of … without affecting how you visit our services by going to ….

Where required by applicable law, we obtain your consent to use cookies.

You may refuse to accept cookies by changing the settings on your device to prevent cookies from being set. However, if you select this setting you may be unable to access certain parts of the System. Unless you have adjusted your browser setting so that it will refuse cookies, our system will issue cookies when you visit the Website and App.

1. **How we use your personal information**

Second Nature is dedicated to maintaining the privacy and integrity of your personal information. As such, we have policies and procedures and other safeguards to help protect your personal information from improper use and disclosure.

We collect and use your personal information to deliver our contract to you. We may collect and use your personal information, including your special category data, only if you have given us your specific consent.

We may use and disclose your personal information and special category data for our internal operations, which include administration, planning and various activities that assess and improve the quality and cost effectiveness of the service that we deliver to you. Examples are using information about you to improve quality of the service, satisfaction surveys, de-identifying personal information, customer services and internal training. We may use and disclose your personal information to contact you as a reminder to interact with, or complete tasks relating to your use of the System.

We use also automated decision-making to allocate you to a group of users before starting the use of our service.

We follow a Minimum Necessary Access Policy, so any required disclosure of your identifiable personal information is minimized. The following categories describe different ways that we use your personal information within Second Nature and disclose your personal information to persons and entities outside of Second Nature. We have not listed every use or disclosure within the categories below, but all permitted uses and disclosures will fall within one of the following categories. In addition, there are some uses and disclosures that may require your specific authorization.

How much personal information is used or disclosed without your written permission will vary depending, for example, on the intended purpose of the use or disclosure.

- — Disclosure at your request: We may disclose personal information relating to your use of the System when requested by you. This disclosure at your request may require written authorization by you.
- — Payment: We do not store your credit/debit card details; they are processed directly by a third party processor (for example, Stripe or Braintree) that will store all payment information and transaction details. We will only retain details of transactions on secure servers and we will not retain your credit or debit card information.
- — Operations: We may use and disclose your personal information for our internal operations, which include administration, planning and various activities that assess and improve the quality and cost effectiveness of the service that we deliver to you. Examples are using information about you to improve quality of the service, satisfaction surveys, de-identifying personal information, customer services and internal training.
- — Reminders and notifications: We may use and disclose your personal information to contact you as a reminder to interact with, or complete tasks relating to your use of the System.
- — Third party service providers:
  - — We may share personal information with third-party service providers we have hired to provide services on our behalf, including those who act as data processors on our behalf. Those data processors are subject to privacy and security obligations consistent with our privacy policy and with the current data protection regulation and framework. They can only use and process the personal information in the ways specified by us. These service providers include (but are not limited to) Amazon Web Services which provides the Second Nature app and website, analytics and search engine providers (including but not limited to Mixpanel, HotJar, Intercom) which assist us in improving Second Nature app or website and your user experience, help us to collect information on your use of the System, and to assist you in case of an issue.
  - — We may share anonymised information with third-party service providers who assist us in our marketing and advertising activities or in the improvement of Second Nature app or website. These are third party services that allow Second Nature to collect information from you concerning your use of the System, including but not limited to pages visited, links clicked, non-sensitive text entered, mouse movements, and usage of our iPhone and Android apps. These services are used to help Second Nature enhance or improve the user experience on this website and to perform any other function that Second Nature reasonably believe in good faith is required to protect and ensure the proper functionality and security of this website.
- — Third party medical professionals: with additional permission that we will separately explain to you and request your consent for, we may disclose your personal information to a third-party medical professional nominated by you: e.g. your GP or local NHS service. This may be in the form of a discharge letter or an electronic disclosure to an electronic patient record.
- — Threat to health or safety: We may use and disclose your personal information when necessary to prevent a serious threat to your health and safety or the health and safety of the public or another person. Any disclosure, however, would only be to someone able to help prevent the threat.
- — As required by law: Certain laws permit or require certain uses and disclosures of personal information, for example, for public health activities, health oversight activities and law enforcement. We may be required to disclose personal information for these and other compliance purposes, including as may be required by applicable laws and regulations or requested by a judicial process or government agency. In these instances, Second Nature will only use or disclose your personal information to the extent the law requires.
- — For research and publicity purposes: We may use personal information for internal and external research and publicity purposes. This may include publishing aggregate, anonymous information about our users in the context of providing public information and conducting academic research.
- — Transfer of business assets: If Second Nature or substantially all of its assets are acquired by a third party, personal information held by it about its customers will be one of the transferred assets.

National personal information opt-out policy: as part as our contracts with the NHS, we collect, process and disclose confidential patient information. We always make sure that we do so for individual care purposes only; we only use and/or disclose anonymised personal information for research purposes, and we don’t use or disclose confidential patient personal information for planning purposes. As such, we are compliant with the National data opt-out policy.

Except as described above, we will never share your personal information with any other party without your consent.

1. **Legal Bases for Processing European Personal Information**

If you are located in the European Economic Area or the United Kingdom, we only process your personal information when we have a valid “legal basis”, including when:

- — Consent. You have consented to the use of your personal information, for example to send you marketing communications or to use cookies.
- — Contractual necessity. We need your personal information to provide you with the services, for example to respond to your inquiries.
- — Compliance with a legal obligation. We have a legal obligation to use your personal information, for example to comply with tax and accounting obligations.
- — Legitimate interests. We or a third party have a legitimate interest in using your personal information. In particular, we have a legitimate interest in using your personal information for product development and internal analytics purposes, and otherwise to improve the safety, security, and performance of our services. We only rely on our or a third party's legitimate interests to process your personal information when these interests are not overridden by your rights and interests.

1. **Where we store your personal information**

All personal information you provide to us is stored on secure servers with trusted 3rd party suppliers, Amazon Web Services ('AWS') within the European Economic Area ('EEA'). AWS complies with the GDPR and the UK GDPR, which set out several data protection requirements, which apply when personal information is being processed. AWS are industry leaders in the provision of hosting services and take security very seriously - you can find out more about their security policies and processes in their [Security Whitepaper](https://d0.awsstatic.com/whitepapers/aws-security-whitepaper.pdf).

We may transfer personal information outside the EEA or the UK to countries deemed adequate by the European Commission; based on Standard Contractual Clauses; to perform the services that you have requested from us, or with your consent.

Unfortunately, despite these measures, the transmission of information via the internet is never completely secure. Although we will do our best to protect your personal information, we cannot guarantee the security of your information transmitted to the System, and any transmission is at your own risk. Once we have received your personal information, we will use strict procedures to try to prevent unauthorized access in accordance with our Company data protection policy and code of practice, and responsibilities as a registered Data Controller in the UK.

1. **Your rights regarding your personal information**

You have certain rights with respect to your personal information. If we do not agree to a request by you with respect to your personal information, please consult the Second Nature Privacy and Security Officer whose contact information is below.

If you are based in the UK or the European Economic Area and we do not comply with any of the below, you have the right to complain to the ICO or to your local Supervisory Authority, and to a judicial remedy.

- — Restrictions: You have the right to request in writing that we do not disclose certain information about you. To request a restriction, please contact the Privacy and Security Officer whose contact information is below.
- — Confidential Communications: You have the right to request in writing that we restrict the way in which we communicate information regarding your health and health care services, such as ceasing to send email or SMS messages to notify or remind you about aspects of the System or your progress through the Second Nature program. We will make every effort to accommodate your request.
- — Access: You have the right to inspect and copy your personal information maintained by us. Normally, we will provide you with access within one month of your request. To request your personal information:
  - — Please download a subject access request form [here](https://docs.google.com/document/d/19e6SQwJq6e7OYcOETXRyWWvyljdvtuzf/), fill it in and return it to us using the contact details on the form, along with copies of information that confirms your identity (if applicable). Please do not send in any original copies of documents. More details of acceptable types of identification documents are included in the application form.
  - — You can email your completed form and electronic copies of your identification documents to: support@secondnature.io
  - — Or print the form, fill it in and post it with paper copies of your identification documents to:
  - — Second Nature
  - — Scale Space White City
  - — 58 Wood Ln
  - — London, W12 7RZ
  - — United Kingdom
  - — We will endeavour to respond promptly and in any event within one month. You can alternatively call us on +44 20 3488 0769
- — Deletion: You have the right to ask that we delete all information that the System has collected on you via email to the Second Nature privacy and Security whose contact information is below.
- — Amendment: You have the right to request that we amend your written personal information. For instance, you can request that we correct an incorrect date of birth in your records. We will amend your personal information within one month of your request, and will notify you when we have amended your personal information. We can deny your request in certain circumstances, such as when we believe that your personal information is accurate and complete.
- — Personal Information Portability: You have the right to obtain and reuse your personal information from Second Nature for your own purposes across different services. This can be freely downloaded in .csv format within the settings page of your Second Nature account.
- — Objection: You have the right to object to processing based on legitimate interests or the performance of a task in the public interest, to direct marketing, and to processing for the purposes of scientific research & statistics. To request an objection, please contact the Privacy and Security Officer whose contact information is below.
- — Automated Decision Making & Profiling: You have the right not to be subject to a decision based on automated processing and it produces a legal effect or a similarly significant effect on you. To request an opt-out of automated decision making & profiling, please contact the Privacy and Security Officer whose contact information is below.

Before meeting your request, we may ask you to provide reasonable information to verify your identity. Please note that there are exceptions and limitations to each of these rights, and that while any changes you make will be reflected in active user databases instantly or within a reasonable period of time, we may retain information for backups, archiving, prevention of fraud and abuse, analytics, satisfaction of legal obligations, or where we otherwise reasonably believe that we have a legitimate reason to do so.

1. **Personal Information Retention**

As per the ICO's 'Principle 5' and the article 5 of GDPR and the UK GDPR, we retain personal information no longer than is necessary for the purpose we obtained it for. With the context that your personal information may be used for research purposes (as covered in section 3), Second Nature will retain any information held on an individual for up to 10 years after that individual has ceased use of the System. At that point, the individual's information will be deleted. As covered in section 5, you may request that we delete your personal information at any time.

1. **EU Representative**

If you are based in the EU Second Nature Healthy Habits Ltd has appointed DataRep as its Data Protection Representative for the purposes of GDPR, so that you can contact them directly in your home country. DataRep has locations in each of the 27 countries and Norway & Iceland in the European Economic Area (EEA). If you want to raise a question to Second Nature Healthy Habits Ltd, or exercise your rights (explained above) in respect of your personal information, you may do so by:

- — Sending an email to DataRep at datarequest@datarep.com, quoting Second Nature Health Ltd in the subject line.
- — Contacting DataRep using their online webform at www.datarep.com/data-request or mailing your enquiry to DataRep at the most convenient of the addresses that you can find below
  - — Austria: DataRep, City Tower, Brückenkopfgasse 1/6. Stock, Graz, 8020, Austria
  - — Belgium: DataRep, Place de L'Université 16, Louvain-La-Neuve, Waals Brabant, 1348, Belgium
  - — Bulgaria: DataRep, 132 Mimi Balkanska Str., Sofia, 1540, Bulgaria
  - — Croatia: DataRep, Ground & 9th Floor, Hoto Tower, Savska cesta 32, Zagreb, 10000, Croatia
  - — Cyprus: DataRep, Victory House, 205 Archbishop Makarios Avenue, Limassol, 3030, Cyprus
  - — Czech: Republic DataRep, IQ Ostrava Ground floor, 28. rijna 3346/91, Ostrava-mesto, Moravska, Ostrava, Czech Republic
  - — Denmark: DataRep, Lautruphøj 1-3, Ballerup, 2750, Denmark
  - — Estonia: DataRep, 2nd Floor, Tornimae 5, Tallinn, 10145, Estonia
  - — Finland: DataRep, Luna House, 5.krs, Mannerheimintie 12 B, Helsinki, 00100, Finland
  - — France: DataRep, 72 rue de Lessard, Rouen, 76100, France
  - — Germany: DataRep, 3rd and 4th floor, Altmarkt 10 B/D, Dresden, 01067, Germany
  - — Greece: DataRep, 24 Lagoumitzi str, Athens, 17671, Greece
  - — Hungary: DataRep, President Centre, Kálmán Imre utca 1, Budapest, 1054, Hungary
  - — Iceland: DataRep, Kalkofnsvegur 2, 101 Reykjavík, Iceland
  - — Ireland: DataRep, The Cube, Monahan Road, Cork, T12 H1XY, Republic of Ireland
  - — Italy: DataRep, BPM 335368, Via Roma 12, 10073 , Ciriè TO, Italy
  - — Latvia: DataRep, 4th & 5th floors, 14 Terbatas Street, Riga, LV-1011, Latvia
  - — Liechtenstein: DataRep, City Tower, Brückenkopfgasse 1/6. Stock, Graz, 8020, Austria
  - — Lithuania: DataRep, 44A Gedimino Avenue, 01110 Vilnius, Lithuania
  - — Luxembourg: DataRep, BPM 335368, Banzelt 4 A, 6921, Roodt-sur-Syre, Luxembourg
  - — Malta: DataRep, Tower Business Centre, 2nd floor, Tower Street, Swatar, BKR4013, Malta
  - — Netherlands: DataRep, Cuserstraat 93, Floor 2 and 3, Amsterdam, 1081 CN, Netherlands
  - — Norway: DataRep, C.J. Hambros Plass 2c, Oslo, 0164, Norway
  - — Poland: DataRep, Budynek Fronton ul Kamienna 21, Krakow, 31-403, Poland
  - — Portugal: DataRep, Torre de Monsanto, Rua Afonso Praça 30, 7th floor, Algès, Lisbon, 1495-061, Portugal
  - — Romania: "DataRep, 15 Piaţa Charles de Gaulle, nr. 1-T, Bucureşti, Sectorul 1, 011857,
  - — Slovakia: DataRep, Apollo Business Centre II, Block E / 9th floor, 4D Prievozska, Bratislava, 821 09, Slovakia
  - — Slovenia: DataRep, Trg. Republike 3, Floor 3, Ljubljana, 1000, Slovenia
  - — Spain: DataRep, BPM 335368, Avd. Castilla La Mancha Nº 70-1 (Nave A), 45270, Mocejon-Toledo, Spain
  - — Sweden: DataRep, S:t Johannesgatan 2, 4th floor, Malmo, SE - 211 46, Sweden

When mailing enquiries, please mark your letters for “DataRep” and not “Second Nature Healthy Habits Ltd”, otherwise the letter may not reach DataRep. Please refer clearly to Second Nature Healthy Habits Ltd in your correspondence. On receiving your correspondence, we are likely to request evidence of your identity, to ensure your personal data and information connected with it is not provided to anyone other than you.

If you have any concerns over how DataRep will handle the personal data they will require to undertake their services, please refer to their privacy policy at [www.datarep.com/privacy-policy](http://www.datarep.com/privacy-policy).

1. **Children's Privacy**

We do not knowingly collect, maintain, or use personal information from children under 13 years of age, and no part of our Service is directed to children. If you learn that a child has provided us with personal information in violation of this Privacy Policy, then you may alert us at [support@secondnature.io](mailto:support@secondnature.io).

1. **Data Security**

We make reasonable efforts to protect your information by using physical and electronic safeguards designed to improve the security of the information we maintain. However, as no electronic transmission or storage of information can be entirely secure, we can make no guarantees as to the security or privacy of your information.

Second Nature provides the System to referrals provided through the NHS. As such, we are compliant with the Data Security and Protection Toolkit 2019 / 2020, our organisation code is 8JF17.

1. **Concerns or complaints**

If you believe that any of your rights with respect to your personal information has been violated by us, our employees or agents, please communicate with the Second Nature Privacy and Security Officer at: support@secondnature.io for UK users, or DataRep for EU-based users (contact details above)

1. **Amending this Policy**

We reserve the right to revise this Policy and to make the revised Policy effective for all personal information that we created or received prior to the effective date of the revised Policy. If you are a registered user, we will notify you of changes by the email address we have for you on file.

Questions relating to revisions to this Policy may be addressed to the Privacy and Security Officer whose contact information is above. This Policy will be promptly revised if there is a material change to a policy described herein.

Effective Date: This Policy is effective as of May 19th 2021.
